# Investigation of the Cell Surface Proteome of Human Periodontal Ligament Stem Cells

**DOI:** 10.1155/2016/1947157

**Published:** 2016-08-04

**Authors:** Jimin Xiong, Danijela Menicanin, Peter S. Zilm, Victor Marino, P. Mark Bartold, Stan Gronthos

**Affiliations:** ^1^Colgate Australian Clinical Dental Research Centre, School of Dentistry, University of Adelaide, Adelaide, SA 50052, Australia; ^2^Laboratory of Tissue Regeneration and Immunology and Department of Periodontics, Beijing Key Laboratory of Tooth Regeneration and Function Reconstruction, School of Stomatology, Capital Medical University, Beijing 1000443, China; ^3^School of Medicine, Faculty of Health Sciences, University of Adelaide, Adelaide, SA 50054, Australia; ^4^Oral Microbiology, School of Dentistry, University of Adelaide, Adelaide, SA 50055, Australia; ^5^Mesenchymal Stem Cell Laboratory, School of Medicine, Faculty of Health Sciences, University of Adelaide, Adelaide, SA 50056, Australia; ^6^South Australian Health and Medical Research Institute, Adelaide, SA 5000, Australia

## Abstract

The present study examined the cell surface proteome of human periodontal ligament stem cells (PDLSC) compared to human fibroblasts. Cell surface proteins were prelabelled with CyDye before processing to extract the membrane lysates, which were separated using 2D electrophoresis. Selected differentially expressed protein “spots” were identified using Mass spectrometry. Four proteins were selected for validation: CD73, CD90, Annexin A2, and sphingosine kinase 1 previously associated with mesenchymal stem cells. Flow cytometric analysis found that CD73 and CD90 were highly expressed by human PDLSC and gingival fibroblasts but not by keratinocytes, indicating that these antigens could be used as potential markers for distinguishing between mesenchymal cells and epithelial cell populations. Annexin A2 was also found to be expressed at low copy number on the cell surface of human PDLSC and gingival fibroblasts, while human keratinocytes lacked any cell surface expression of Annexin A2. In contrast, sphingosine kinase 1 expression was detected in all the cell types examined using immunocytochemical analysis. These proteomic studies form the foundation to further define the cell surface protein expression profile of PDLSC in order to better characterise this cell population and help develop novel strategies for the purification of this stem cell population.

## 1. Introduction 

Despite encouraging outcomes, therapeutic utilization of mesenchymal stem cells (MSC) is constrained by the lack of understanding and definition of their properties and developmental status following* ex vivo* expansion. Heterogeneity inherent within progenitor populations presents as one of the major limitations to their clinical application in regenerative medicine. The variability and inconsistencies in cellular properties allude to a hierarchical order within stem cell populations and result in the coexistence of subsets of distinct morphologies, phenotypes, proliferation rates, and biological functions [1–3]. Currently, there is a lack of individual or a set of markers that can distinguish different subsets within MSC-like populations of different origins from more differentiated fibroblastic cells in any tissue.

Identification of stem/progenitor cells residing in the periodontium [4–6] has offered a potential novel therapeutic avenue for treating periodontal tissues damaged due to trauma, injury, and disease. Periodontal diseases are highly prevalent among all human populations and if untreated cause the destruction of periodontal supporting tissues and can potentially result in tooth loss. Predictable regeneration of periodontal tissues as a result of advanced periodontal diseases is beyond the scope of current technologies and, therefore, alternative strategies are being investigated.

In addition to periodontal ligament stem cells (PDLSC), the periodontium contains multiple cell types including fibroblasts, endothelial cells, epithelial cell rests of Malassez (ERM), osteoblasts, and cementoblasts [7]. This array of specialised cell types is integrated into and cofunctions to provide the periodontium with its essential and unique structural and mechanical properties. This biological complexity and cellular heterogeneity highlights the need for identification of surface markers specific to each cell subset within the periodontium to enable identification and discriminant isolation of desired and required cell populations.

It has been demonstrated that PDLSC share a phenotypic profile characteristic of bone marrow derived mesenchymal stem cells (BMSC) including expression of BMSC markers CD29, CD44, CD90, and CD105 [8]. Furthermore, PDLSC express the early BMSC and perivascular cell surface markers STRO-1 and CD146/MUC18 [4], with a subset of progenitors presenting with other antigens associated with perivascular tissues (alpha-smooth muscle actin and pericyte-associated antigen, 3G5) [9]. Together, these findings designate a possible perivascular origin of PDLSC, in accord with earlier findings by McCulloch and colleagues [10, 11]. In conjunction, comparative genomic analyses identified unique features exhibited by PDLSC when compared to BMSC and dental pulp stem cells (DPSC). These studies demonstrated increased levels of scleraxis (a tendon-specific transcription factor) [4] and PLAP-1 (periodontal ligament associated protein-1/asporin) expression in PDLSC [12]. A panel of markers, proposed for the current identification of PDLSC, includes alkaline phosphatase, type I collagen, periostin, runt-related transcription factor-2 (Runx2), and epithelial growth factor receptor, which are also expressed by BMSC, considering that both cell populations commonly hold the innate capacity for formation of mineralized matrix in the form of cementum and bone, respectively [13]. Since the cell surface markers described above are ubiquitously expressed by MSC-like populations derived from all dental tissues, specific cell surface antigens, capable of distinguishing between individual dental stem cell population subsets, are yet to be identified [14]. Therefore, our understanding of the cell surface phenotype of PDLSC falls short when considering the need to isolate and purify stem/progenitor cell subsets from the heterogeneous PDL population. This has driven the use of proteomics, the technology investigating global protein expression, to characterise the cell surface phenotype of PDLSC.

Proteomic studies investigating dental tissues have been summarized by McCulloch [15]. While the majority of studies focused on protein expression by periodontal microbiota [16–18], a limited number of papers examined proteomic profiles of periodontal ligament cells and tissues [15]. In this study, we provide an insight into the cell surface proteome of PDLSC to identify potential discriminatory PDLSC markers not expressed by other cells residing in the periodontium.

## 2. Methods and Materials

### 2.1. Isolation of Human PDLSC and Gingival Fibroblasts

Human PDLSC and gingival fibroblasts (GF) were isolated from three donors and cultured as previously described (Human Research Ethics Committee of the University of Adelaide, Approval Number H-112-2008) [4, 19]. Briefly, gingival and periodontal ligament tissues were collected from excised gingiva and middle third of the root, respectively. The tissues were digested in equal volumes of collagenase type I (3 mg/mL; Worthington Biochemical, Lakewood, NJ) and dispase type II (4 mg/mL; Roche Diagnostics, Indianapolis, IN) for 2 hours at 37°C. Isolated cells were maintained and cultured in modified *α*-MEM media (*α*-MEM; Sigma-Aldrich, St. Louis, MO, USA) supplemented with 10% FCS (Thermo Electron, Melbourne, VIC, Australia), 50 U/mL and 50 *μ*g/mL penicillin and streptomycin (Sigma-Aldrich), 1 mM sodium pyruvate, 2 mM L-glutamine (SAFC, Lenexa, KS, USA), and 100 *μ*M L-ascorbate-2-phosphate (Novachem, Melbourne, VIC, Australia) at 37°C and 5% CO_2_ in a humidified environment, with a twice-weekly medium change. Cells were harvested and further expanded once upon reaching confluence. This process was repeated when cells reached 80% confluence until desired cell numbers were obtained.

Human neonatal foreskins, collected from routine circumcisions, were used to isolate epithelial sheets after overnight incubation with 4 mg/mL dispase at 4°C, followed by trypsinization for 5 min at 37°C to obtain basal keratinocytes. Keratinocytes were cultured in DMEM containing 10% fetal calf serum, 20 ng/mL epidermal growth factor (Sigma-Aldrich), and 0.4 *μ*g/mL hydrocortisone (Sigma-Aldrich), at 37°C and 5% CO_2_ in a humidified environment, with a twice-weekly medium change.

### 2.2. Immunohistochemistry

Chamber slides (Nalge-Nunc Lab-Tek, Rochester, NY, USA) were seeded with 8 × 10^3^ cells per cm^2^, in media with additives for 2 days. The slides were fixed with 4% paraformaldehyde (PFA) and endogenous peroxidase activity was inhibited using 0.5% H_2_O_2_ in methanol. The sections were incubated with primary antibodies or isotype control antibodies overnight at 4°C, secondary antibodies for 1 hour at room temperature, Vectastain ABC Reagents (Vector Laboratories, Burlingame, CA, USA) according to the manufacturer's recommendations, or horseradish-peroxidase-labelled streptavidin (Promega, Madison, WI, USA) at 1 in 1000 dilution and then developed with diaminobenzidine (Dako, Campbellfield, VIC, Australia). The slides were counterstained briefly with haematoxylin (ProSciTech, Thuringowa Central, QLD, Australia). Antibodies used in this study are 1B5, mouse IgG1 isotype control (1 : 25; Professor L. K. Ashman, University of Newcastle, NSW, Australia); mouse IgG1 anti-human CD73 (1 : 25; BD Pharmingen, Sparks, MD, USA); mouse IgG1 anti-human CD90 (1 : 25; BD Pharmingen); mouse IgG1 anti-human Annexin A2 (1 : 12.5; Invitrogen, Waltham, MA, USA); rabbit anti-human sphingosine kinase 1 (1 : 20; Cayman Chemical, Ann Arbor, MI, USA); normal rabbit Ig (1 : 20; Vector Laboratories, Burlingame, CA, USA); goat anti-mouse IgG biotin secondary antibody (1 : 200; Southern Biotechnology Associates, Birmingham, AL); and goat anti-rabbit IgG biotin secondary antibody (1 : 150; Vector Laboratories).

### 2.3. Immunophenotypic Profiling

Single cell suspensions of 2 × 10^5^ cells were blocked with 5% FCS, 1% bovine serum albumin (BSA, SAFC), 50 U/mL penicillin, 50 *μ*g/mL streptomycin, and 5% normal human serum (Red Cross, SA, Australia) in HBSS on ice. Cells were treated with primary or isotype control antibodies (CD73, CD90, and Annexin A2) at a concentration of 20 *μ*g/mL, followed by incubation with phycoerythrin (PE) conjugated goat anti-mouse IgG1 (1 : 50; Southern Biotechnology Associates). Samples were fixed in PBS with 0.1% formalin and 20 mg/mL glucose. Analysis was performed on a fluorescence-activated cell sorter fitted with 250 MW argon laser (Beckman Coulter Cytomics FC500, using CXP Cytometry List Mode Data Acquisition and Analysis Software version 2.2; Beckman Coulter, Miami, FL, USA).

### 2.4. Proteomic Analysis

All equipment and reagents were purchased from Bio-Rad Laboratories (Hercules, CA, USA) unless stated otherwise. CyDye DIGE Fluor minimal dye was purchased from GE Healthcare (Buckinghamshire, UK).

### 2.5. Cell Surface Labelling Using CyDye DIGE Fluor Minimal Dye

CyDye fluorescent cell surface protein labelling was performed as previously reported [4, 20]. Briefly, approximately 20 million subconfluent PDLSC or GF were detached with either 1 mM PUCK's EDTA or 3 mg/mL type I collagenase and aliquoted into ~5 million cells per tube. Cells were washed in ice cold HBSS (pH 7.4) followed by ice cold HBSS (pH 8.5) and centrifuged at 800 ×g for 2 minutes. The cell pellets were resuspended in 200 *μ*L labelling buffer containing HBSS (pH 8.5) and 1 M urea. Cells were then labelled with 600 pmol of either Cy2, Cy3, or Cy5 or CyDye DIGE Fluor minimal dyes on ice in the dark for 20 minutes. Staining was quenched by adding 20 *μ*L lysine (10 mM) for 10 minutes. Surface-labelled cells were pelleted by centrifugation and resuspended in 202 *μ*L HBSS (pH 7.4). An aliquot (2 *μ*L) was taken prior to and after labelling to check for labelling efficiency using flow cytometry.

### 2.6. Membrane Protein Enrichment

Proteins were isolated and fractionated using a phase separation kit (Mem-PER, Pierce, Rockford, IL, USA) according to the manufacturer's instructions. Briefly, 150 *μ*L reagent A containing 1 *μ*L protease inhibitor (Sigma) was added to cell pellets containing PDLSC or GF. Following 10-minute incubation, 450 *μ*L mixture of reagents B and C was added to cell lysates and tubes were kept on ice for 30 minutes. The preparation was centrifuged at 10,000 ×g for 3 minutes, at 4°C, and the supernatant was incubated at 37°C for 20 minutes. Following centrifugation at 10,000 ×g and phase separation, the hydrophobic fraction containing membrane proteins was carefully removed and purified using ReadyPrep 2-D Cleanup Kit. Membrane protein enrichment efficiency was assessed and cells were subjected to up to three membrane fractionation steps.

### 2.7. Membrane Protein Separation by Two-Dimensional Gel Electrophoresis (2DE)

Membrane proteins were solubilised with ReadyPrep reagent 3 buffer for 1 hour at room temperature. The protein was solubilised by gentle aspiration through a fine-gauge needle, as previously described by Zilm et al. [21]. The protein concentration was determined using RC/DC Protein Assay Kit according to the manufacturer's instructions. Proteins were separated in the first dimension using 11 cm immobilised pH gradient (IPG) strips (pH 3–10) which had been passively rehydrated for 24 hours in 330 *μ*L rehydration/extraction buffer #3, containing 0.2% (w/v) pH 3–10 ampholytes and 1.2% (v/v) De-Streak Reagent (GE Healthcare, Buckinghamshire, UK). IEF was performed using a Protean IEF cell. Briefly, membrane protein preparations containing 150 *μ*g protein were cup-loaded onto the anode end of the IPG strip. The IEF cycle consisted of 8 steps outlined in Table 1, with a 50 *μ*A/strip current limit, and the temperature was maintained at 20°C. Duplicate IPG strips were run concurrently. Following IEF, the IPG strips were equilibrated as previously described [22]. Polyacrylamide gels (18 × 18 cm) containing 8% T, 3.3% C, 0.1% SDS, and 375 mM Tris/HCl (pH 8.8) were cast without stacking gels using a Protean II XL casting chamber. Proteins were separated in the second dimension using a Protean II XL Multicell (Bio-Rad Laboratories) in tris-glycine tank buffer (25 mM Tris, 192 mM glycine, and 0.1% (w/v) SDS) and resolved at 7 mA/gel.

### 2.8. Gel Visualisation

Gels were scanned using a Typhoon Trio Variable Mode Imager (Molecular Dynamics Inc., Sunnyvale, CA) with a pixel resolution of 100 *μ*m at the CyDye excitation and emission wavelengths described by the manufacturer. Image analysis was performed using PD-Quest software (version 7.2, Bio-Rad Laboratories). Replicate groups, each containing four gels, were used for analysis. Protein spots were automatically detected and manually edited. Gel staining was normalized using the total density in gels.

### 2.9. Flamingo Fluorescent Staining

To visualise all proteins, gels were fixed in 40% ethanol (v/v)/10% acetic acid (v/v) in Milli-Q water and stained with Flamingo Fluorescent Stain (Bio-Rad Laboratories) according to the manufacturer's instructions. Gels were destained in 0.1% (v/v) Tween 20 in Milli-Q water for 10 minutes prior to imaging. Gels were scanned using a Typhoon Trio Variable Mode Imager using a green laser (532 nm) excitation source and 610 ± 30 nm bandpass emission filter.

### 2.10. Automated Spot Picking

Gel images were scanned using a Typhoon Trio Variable Mode Imager and imported into DeCyder software (version 6.5, GE Healthcare) and spots were detected using the automated method. Spots of interest were selected to generate a pick-list. The pick-list was exported from DeCyder and imported into Spot Picker software (version 1.2, GE Healthcare). Spots were excised using the Ettan Spot Cutting Robot (GE Healthcare) according to the manufacturer's instructions. Gel plugs were washed twice with 0.1 M ammonium bicarbonate buffer (NH_4_HCO_3_), followed by Milli-Q water, then dehydrated in acetonitrile (ACN), and dried.

### 2.11. Protein Identification by Liquid Chromatography-Electrospray Ionisation-Ion Trap (LC-ESI-IT) Mass Spectrometry (MS)

Each gel plug was digested with 10 *μ*L of 5 mM ammonium bicarbonate with 10% ACN containing 100 ng trypsin (Promega) for 16 hours at 37°C. Peptides were extracted sequentially with 1% formic acid (FA), 50% ACN/0.1% FA, and ACN, and the combined extracts were concentrated by centrifugal evaporation and diluted in 6 *μ*L 3% ACN/0.1% FA. Vacuum concentrated samples were resuspended in 0.1% FA in 2% ACN to a total volume of ~8 *μ*L. LC-ESI-IT MS/MS was performed using an online 1100 series HPLC system (Agilent Technologies) and HCT Ultra 3D-Ion Trap mass spectrometer (Bruker Daltonics). The LC system was interfaced to the MS using an Agilent Technologies Chip Cube operating with a ProtID-Chip-150 (II), which integrates the enrichment column (Zorbax 300SB-C18, 4 mm, 40 nL), analytical column (Zorbax 300 SB-C18, 150 mm × 75 *μ*m), and nanospray emitter. 5 *μ*L samples were loaded onto the enrichment column set at a flow rate of 4 *μ*L/min in Mobile Phase A (0.1% FA in 2% v/v ACN) and resolved with 1–30% gradient of Mobile Phase B (0.1% FA in 98% w/v ACN) over 32 minutes at 300 nL/min. Ionizable species (300 <* m/z *< 3,000) were trapped and the two most intense ions eluting at the time were fragmented by collision-induced dissociation. Active exclusion was used to exclude a precursor ion for 30 seconds following the acquisition of two spectra.

### 2.12. Protein Identification Using Web-Based Bioinformatics Tools

MS and MS/MS spectra were subjected to peak detection and deconvolution using Data Analysis (version 3.4, Bruker Daltonics, Billerica, MA, USA). Compound lists were exported into BioTools (version 3.1, Bruker Daltonics) and then submitted to Mascot (version 2.2, Boston, MA, USA) using the following parameters: fixed modification = carbamidomethyl (C), variable modification = oxidation (M), MS mass tolerance = 1.5 Da, MS/MS mass tolerance = 0.8 Da, peptide charge = 1+, 2+, or 3+, and missed cleavages = 3. Data were matched to the Swiss-Prot protein database.

## 3. Results

### 3.1. Membrane Protein Expression of* Ex Vivo* Expanded Human PDLSC

**Figure 1:**
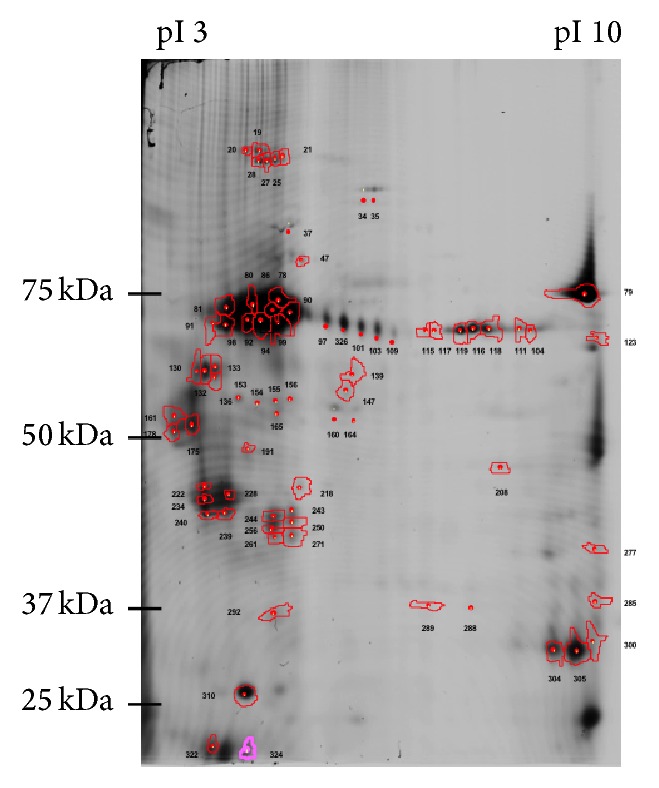
Representative raw 2DE gel of CyDye labelled proteins of* ex vivo* expanded human periodontal ligament stem cells and the location of proteins identified on the raw 2DE image. Following cell surface labelling with CyDye and membrane protein enrichment, proteins were separated by 2DE using a pI range of pH 3–10 and a molecular weight range of 10–110 kDa. Following image analysis, 80 well-resolved protein spots were detected. A total of 32 membrane-associated protein spots were consistently found on replicate gels.

CyDye-tagged membrane-associated proteins derived from human PDLSC following* ex vivo* expansion were separated by 2-dimensional electrophoresis. Based on the CyDye imaging, a total of 80 well-resolved proteins spots with a molecular weight range of 10–110 kDa were detected after automatic exclusion of pseudospots and the locations of the identified protein spots on the representative raw image are shown in Figure 1.

### 3.2. Identification of Proteins Expressed by Human PDLSC

Following spot excision and analysis by mass spectrometry, a total of 32 protein spots were identified as membrane-associated proteins (Figure 1).  Table 2 outlines the details of the membrane-associated proteins, including the protein name, spot number, predicted molecular weight and pI values, ID/total queries, combined ion scores, and coverage. Some proteins were identified in multiple spots (e.g., 5′-nucleotidase, Annexin A2, and sphingosine kinase 1) suggesting the presence of isoforms, possibly as a result of posttranslational modifications. Differences in the observed molecular weight/pI and the expected values were observed in some proteins (e.g., sphingosine kinase 1), possibly due to posttranslational modifications, proteolysis, or protein aggregation. Importantly, this approach was validated by the identification of MSC-associated stem cell surface proteins, 5′-nucleotidase (CD73) and Thy-1 membrane glycoprotein (CD90), previously shown to be expressed by PDLSC [8]. Furthermore, MS identified other membrane-associated markers, such as Annexin A2 and sphingosine kinase 1, the expression of which had not previously been reported by human PDLSC. All four proteins were chosen for further confirmatory analyses.

### 3.3. Validation of the Expression of 5′-Nucleotidase, Thy-1 Membrane Glycoprotein, Annexin A2, and Sphingosine Kinase 1

**Figure 2:**
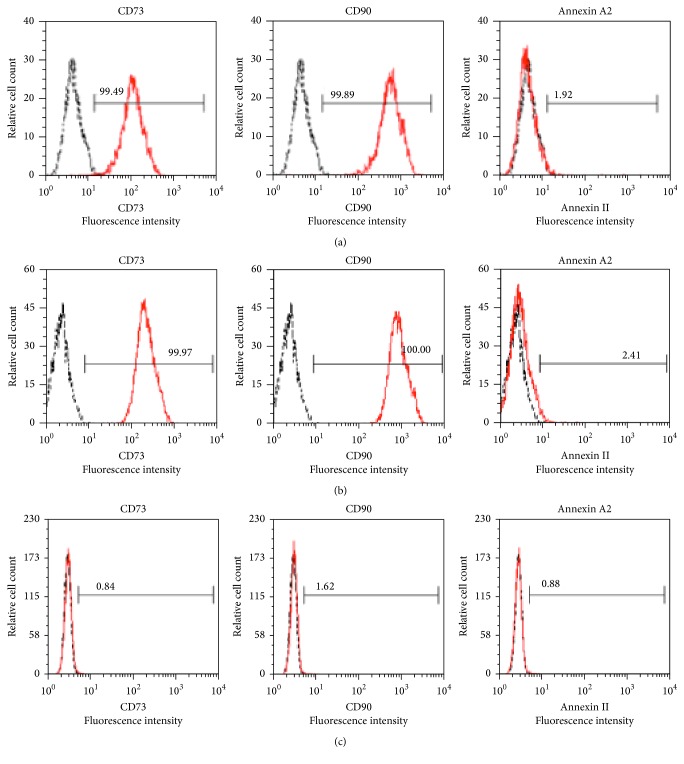
Validation of surface expression of CD73, CD90, and Annexin A2 using flow cytometric analysis in human (a) periodontal ligament stem cells, (b) gingival fibroblasts, and (c) skin keratinocytes.

To confirm the expression of selected proteins including CD73, CD90, Annexin A2, and sphingosine kinase 1 (SPK1), additional studies were performed to investigate their expression in human PDLSC, GF, and keratinocytes (epithelial cell population). Flow cytometric analysis demonstrated high surface expression of CD73 and CD90 and low cell surface levels of Annexin A2 expression in human PDLSC and GF populations (Figure 2). In contrast, human keratinocytes showed a lack of cell surface expression for CD73, CD90, and Annexin A2 (Figure 2).  Table 3 summarizes levels of surface expression of these four antigens on assessed cell types. In summary, CD73 and CD90 were expressed by human PDLSC and GF, but not by human keratinocytes, confirming that they are MSC-associated markers. Annexin A2 was demonstrated to be expressed at low levels by human PDLSC (1.92–7.83%) and human GF (2.41–4.66%), while human keratinocytes were largely negative for Annexin A2 expression (0.88–1.64%). Previous studies have shown that SPK1 can translocate to the plasma membrane upon cell stimulation by cytokines [23–29]. No positive expression was detected with the anti-SPK1 antibody to the human cell types by flow cytometric analysis (data not shown), most likely because the available antibody reagent did not react with the extracellular domain of SPK1.

**Figure 3:**
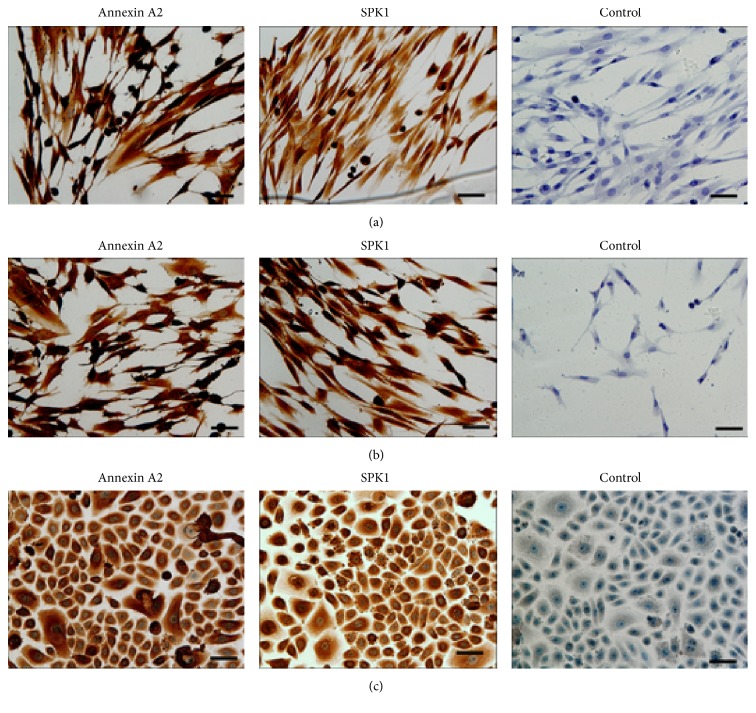
The expression of Annexin A2 and sphingosine kinase 1 (SPK1) in fixed and permeabilized (a) human periodontal ligament stem cells (PDLSC), (b) gingival fibroblasts (GF), and (c) keratinocytes using immunohistochemistry. All cell types studied were positive to anti-Annexin A2 and anti-SPK1 antibodies. Scale bar = 50 *μ*m.

Additional studies were performed to investigate the expression of Annexin A2 and SPK1 in human PDLSC, GF, and keratinocytes, using immunocytochemistry. All cell types studied were positive to anti-Annexin A2 and anti-SPK1 antibodies (Figure 3). Of note, no reactivity was observed with the anti-CD73 or anti-CD90 antibodies to all cell types examined (data not shown), indicating that the specific epitopes identified by these antibodies were compromised following processing for immunocytochemical analysis.

## 4. Discussion 

Initially, the first proteomic reference map of undifferentiated periodontal ligament fibroblasts identified 117 proteins, consistently expressed across three clones, which included a variety of expected cytoskeleton- and metabolism-related proteins [30]. This comparative analysis of the proteome revealed that the percentage of total cytoskeleton-related proteins identified in periodontal ligament fibroblasts (26.5%) was higher than that in dermal fibroblasts (15%). It was proposed that this difference is assigned to mechanical loading and rapid remodelling associated with periodontal ligament tissue [30].

Assessment of protein expression during differentiation of PDLSC identified 29 proteins, differentially expressed during early cementoblastic/osteogenic differentiation [31], and demonstrated a reduction in expression of cytoskeletal proteins and their binding partners, potentially attributed to cytoskeletal rearrangements during differentiation processes [32]. Interestingly, higher expression of the calcium-binding protein Annexin A4 was noted following osteogenic differentiation. Annexins are thought to play an important role in osteogenic development including Annexin A2 and Annexin A5 which are highly expressed in skeletal tissues and upregulated in osteogenic cultures of MSC [33, 34].

A direct comparison of protein expression profiles between ovine PDLSC, DPSC, and BMSC identified 58 differentially expressed proteins between at least two MSC populations, with the expression of 6 proteins upregulated in PDLSC relative to both DPSC and BMSC, 5 proteins upregulated in DPSC relative to both PDLSC and BMSC, and 1 protein upregulated in BMSC relative to both PDLSC and DPSC [35]. An increase in PDLSC expression of heat-shock protein beta 1, Annexin A3, and Annexin A4 compared to DPSC and BMSC was thought to relate to high turnover of periodontal tissues.

The aim of the present study was to determine the surface expression profile of human PDLSC and to compare the expression of prospective cell surface markers in human PDLSC, GF, and keratinocytes (as a source of epithelial cells). Our findings identified 80 proteins expressed on the surface of human PDLSC, 32 of which were membrane associated and four of which were selected for further validation due to their known association with other MSC-like populations as a proof-of-principal analysis. These include CD73 and CD90, well known MSC-associated markers, and Annexin A2 and SPK1. Annexin A2 is calcium dependent [36–42] and has been reported to be associated with the stem cell niche [43–49] and SPK1 has recently been demonstrated to be associated with the progenitor phenotype of endothelial cells [50]. CD73 and CD90 were highly expressed by human PDLSC and GF but not by human keratinocytes, indicating that these antigens could be used as potential markers for distinguishing mesenchymal from epithelial cell populations. Annexin A2 was demonstratively expressed at the cell surface at low copy number by human PDLSC and GF, using flow cytometric analysis, while human keratinocytes lacked any cell surface expression of Annexin A2. Expression of SPK1 was detected in all analysed cell types using immunocytochemical analysis.

CD73, originally defined as a lymphocyte differentiation antigen, functions as a cosignalling molecule on T lymphocytes and is required for lymphocyte binding to endothelium [51]. Expression of CD73 has been demonstrated on various cell types including lymphocytes, endothelial cells, and MSC. It is thought to play physiological roles in epithelial ion and fluid transport, maintaining barrier functions, mediating endothelial permeability, adapting to hypoxia, and contributing to microbial responses [52]. CD73 is an extracellular enzyme that catalyzes the formation of immunosuppressive adenosine by converting adenosine 5′-monophosphate (AMP) to its bioactive intermediate, adenosine, which in turn activates adenosine receptors, when released into the extracellular space, and regulates various physiological functions [52, 53]. Adenosine signalling, modulated by CD39 and CD73 expression, has been highlighted as a novel modulator in the immunosuppression of T-cell proliferation by MSC [54, 55]. As such, this may be contributory to immunomodulatory properties of PDLSC [8] and may highlight an avenue for anti-inflammatory therapy in periodontal disease [4, 56].

The expression of CD90 on PDLSC has been well documented [56–59]; however, its role in PDLSC function remains largely unknown. CD90, also known as Thy-1 (thymocyte differentiation antigen-1), is found to be expressed in various cell types such as hematopoietic stem/progenitor cells [60], hepatic stem cells in human fetal liver [61], liver cancer stem cells [62], neurons, fibroblasts, vascular pericytes, and MSC [63]. Its expression is developmentally regulated [64] and remains one of the minimal criteria for defining human MSC, proposed by the committee for the International Society for Cellular Therapy (ISCT) [63, 65]. While the biological role of CD90 is unclear, a number of associated immunological and nonimmunological functions have been previously addressed [64]. In addition to its involvement in T-cell activation [64], it is believed to be associated with many cellular processes and pathological conditions in a context-dependent manner [63], including cell-cell and cell-matrix interactions, cell motility, and thymocyte adhesion to epithelium [64]. Moreover, CD90 expression is also associated with fibroblast phenotypes relevant to wound healing and fibrosis. The differential CD90 expression is associated with cell-extracellular matrix interactions and cell migration and, as such, is correlated with distinct cellular morphology [64].

Annexin A2, firstly identified as an intracellular protein, has since been found extracellularly both in secreted and in membrane bound form [66]. While Annexin A2 monomer is largely present in the cytoplasm, the formation of the heterotetramer allows its binding to the plasma membrane [67–70]. Potential roles of extracellular Annexin A2 include plasminogen activation, cell-cell adhesion, and immunoglobulin transport [66]. Increasing evidence has highlighted the roles of the Annexin family of calcium-dependent, phopholipid-binding proteins in the mineralization process [36–42] and found them to be highly expressed in calcifying cartilage and bone and to serve to initiate mineralization of extracellular matrix [71]. Previously, it has been suggested that Annexin members also have the capacity to function in a compensatory manner of each other during skeletal development [72]. In a study investigating intracellular processes involved in mineralization, the overexpression of Annexin A2 has been shown to increase ALP activity and cartilage and bone formation, while diminished Annexin A2 expression resulted in decreased mineralization [38]. Collectively, previous findings related to Annexins in dental tissues are consistent with their roles in support of osteogenic differentiation and formation of minerals [31, 33, 35, 73, 74].

We identified Annexin A2 as one of the cell surface proteins expressed by human PDLSC. Further flow cytometric analysis showed the surface expression of Annexin A2 at low copy number by human GF. As a member of the Annexin family which plays important roles in the mineralization process [36–42], the low expression of Annexin A2 in human GF may be correlated to the fact that human GF demonstrated limited osteogenic potential when cultured in osteogenic conditions (data not shown). A recent study [75] suggested that Annexin A2 regulates adhesion, homing, and engraftment within stem cell niches at endosteal [43–47] and vascular [48, 49] sites; hence, we propose that it may be a potential marker of the PDLSC niche in periodontal tissues.

SPK1, the more characterised of the two SPK isoforms, enhances cell growth and proliferation and is involved in immune regulation and tumorigenesis [76]. This highly conserved lipid kinase catalyzes the phosphorylation of proapoptotic sphingosine to form antiapoptotic sphingosine-1-phosphate (S1P) [77] and, as such, is an important cell fate determinant [78]. S1P is a key sphingolipid metabolite that regulates various physiological and pathological processes such as cell proliferation, differentiation, apoptosis, migration, invasion, and angiogenesis [78] and is thought to promote cell growth and proliferation and suppresses apoptosis [79]. In addition to their roles in regulating cell proliferation and apoptosis, SPK-S1P-S1P receptors have been shown to be involved in immune regulation such as immune cell trafficking, activation, and T-cell differentiation [77].

Multiple studies have demonstrated that SPK and S1P play important roles in the maintenance of stem cells [76] including endothelial progenitor cells, rate of endothelial progenitor cell differentiation [50, 80, 81], neural progenitors [82], human embryonic stem cells [76, 83], hematopoietic stem cell [76], and muscle progenitors [84]. Furthermore, SPK1 is a maker of oncogenic potential, tumour progression, and cancer prognosis in numerous tissue types [85, 86]. It is predominantly a cytosolic enzyme, which lacks an obvious membrane anchoring sequence. However, considerable evidence has suggested that SPK1 can be translocated to the plasma membrane upon cell stimulation by growth factors and cytokines [23–25, 27–29, 83]. The present study initially identified SPK1 in human PDLSC by proteomic analysis and SPK1 expression was demonstrated in all cell types examined by immunocytochemical analysis. However, we were unable to demonstrate the cell surface expression of this enzyme, limited by the availability of an SPK1 antibody suitable for flow cytometry.

## 5. Conclusion

In summary, this study is the first, to date, to investigate the cell surface proteome of* ex vivo* expanded human PDLSC. In addition to the expression of recognised MSC-associated cell surface antigens CD73 and CD90, PDLSC were also found to express two novel cell surface proteins, Annexin A2 and sphingosine kinase 1. Interestingly, previous studies have implicated CD73, CD90, Annexin A2, and sphingosine kinase 1 expression in the maintenance of various stem cell populations. Importantly, this study found that human skin epithelial cells lacked the expression of CD73, CD90, and Annexin A2. These proteomic findings provide the platform to further define the cell surface protein expression profile of PDLSC in order to further characterise this cell population and support development of novel isolation and purification strategies.

## Figures and Tables

**Table 1:** IEF conditions for 11 cm IPG (3–10).

Step number	Voltage	Voltage ramping mode	Time
Step 1	150 V	Linear	1 hour
Step 2	300 V	Linear	2.30 hours
Step 3	600 V	Linear	1.50 hours
Step 4	1200 V	Linear	1.50 hours
Step 5	4000 V	Slow	1.50 hours
Step 6	8000 V	Slow	1 hour
Step 7	8000 V	Linear	30000 volt-hours
Step 8	500 V	Slow	0.15 hours

**Table 2:** Membrane-associated proteins on human PDLSC.

Swiss-Prot 57.7 Accession number	Protein name	Spot number	Predicted MW (kDa)/pI	ID/total queries	Combined ion scores	Coverage (%)
1A01_HUMAN	HLA class I histocompatibility antigen, A-1 alpha chain	243	41.1/6.1	2/612	66	10

1C06_HUMAN	HLA class I histocompatibility antigen, Cw-6 alpha chain	239	41.4/5.7	4/638	175	18

5NTD_HUMAN	5′-Nucleotidase	78	63.9/6.6	13/518	287	17
		80	63.9/6.6	8/513	206	11
		90	63.9/6.6	41/546	872	32
		99	63.9/6.6	13/535	191	15
		101	63.9/6.6	39/517	945	38
		103	63.9/6.6	42/539	933	33
		109	63.9/6.6	25/536	606	22
		111	63.9/6.6	2/558	34	6
		326	63.9/6.6	53/544	961	35
		86	63.9/6.6	25/356	521	31
		97	63.9/6.6	45/414	1044	39

AMPB_HUMAN	Aminopeptidase B	94	73.2/5.5	5/559	172	10

ANXA2_HUMAN	Annexin A2	285	38.8/7.6	36/581	489	47
		288	38.8/7.6	87/585	1907	67
		289	38.8/7.6	56/600	950	55
		292	38.8/7.6	3/594	76	11
		304	38.8/7.6	14/536	241	28

CAP2_HUMAN	Adenylyl cyclase-associated protein 2	154	53.1/6.0	2/576	67	6
		156	53.1/6.0	4/557	102	8
		165	53.1/6.0	3/619	102	11

CO1A1_HUMAN	Collagen alpha-1(I) chain	292	139.9/5.6	7/594	103	4

CO6A3_HUMAN	Collagen alpha-3(VI) chain	20	345.2/6.3	9/568	159	3

DNJA1_HUMAN	DnaJ homolog subfamily A member 1	208	45.6/6.6	4/521	95	12

EHD3_HUMAN	EH domain-containing protein 3	139	62.0/6.1	4/600	71	8

EZRI_HUMAN	Ezrin	65	69.5/5.9	28/558	455	26

FLNC_HUMAN	Filamin-C	29	293.4/5.6	4/492	49	2
		78	293.4/5.6	3/518	65	1
		139	293.4/5.6	14/600	363	6
		147	293.4/5.6	13/525	237	4
		191	293.4/5.6	4/586	147	2

GELS_HUMAN	Gelsolin	46	86.0/5.9	46/636	1011	35

K2C1_HUMAN	Keratin, type II cytoskeletal 1	25	66.2/8.2	5/578	128	9
		46	66.2/8.2	11/636	456	17
		47	66.2/8.2	7/491	170	7
		80	66.2/8.2	11/513	227	13
		91	66.2/8.2	3/666	94	12
		94	66.2/8.2	4/559	146	8
		98	66.2/8.2	2/639	57	7
		104	66.2/8.2	2/520	80	3
		111	66.2/8.2	5/558	79	10
		130	66.2/8.2	3/614	60	8
		147	66.2/8.2	20/525	256	13
		153	66.2/8.2	2/615	126	7
		154	66.2/8.2	3/576	107	8
		228	66.2/8.2	10/664	466	22
		289	66.2/8.2	14/600	386	15
		292	66.2/8.2	2/594	55	3
		324	66.2/8.2	5/570	127	10

KAP2_HUMAN	cAMP-dependent protein kinase type II-alpha regulatory subunit	178	45.8/5.0	13/621	360	22

NOMO2_HUMAN	Nodal modulator 2	19	140.4/5.5	9/599	244	8
		20	140.4/5.5	10/568	189	12
		25	140.4/5.5	10/578	226	8
		27	140.4/5.5	11/583	224	10
		28	140.4/5.5	11/436	244	12

NUCB2_HUMAN	Nucleobindin-2	178	50.3/5.0	3/621	100	5

PDIA6_HUMAN	Protein disulfide-isomerase A6	175	48.5/5.0	6/592	209	12
		178	48.5/5.0	7/621	278	10

RUVB2_HUMAN	RuvB-like 2	191	51.3/5.5	25/586	614	34

SBP1_HUMAN	Selenium-binding protein 1	165	52.9/5.9	7/619	126	8

SNX4_HUMAN	Sorting nexin-4	155	52.2/5.7	5/637	157	14

SPHK1_HUMAN	Sphingosine kinase 1	19	42.9/6.6	12/599	253	18
		20	42.9/6.6	5/568	188	16
		21	42.9/6.6	4/518	138	11
		34	42.9/6.6	3/517	200	11

STML2_HUMAN	Stomatin-like protein 2	239	38.6/6.9	13/638	584	37
		240	38.6/6.9	6/656	266	27

STXB3_HUMAN	Syntaxin-binding protein 3	111	68.6/8.0	5/558	97	7
		118	68.6/8.0	6/479	103	7

SWP70_HUMAN	Switch-associated protein 70	94	69.4/5.7	3/559	80	5
		99	69.4/5.7	6/535	136	7

THY1_HUMAN	Thy-1 membrane glycoprotein	304	18.2/9.0	3/536	55	16

UBP14_HUMAN	Ubiquitin carboxyl-terminal hydrolase 14	130	56.5/5.2	6/614	176	15
		132	56.5/5.2	8/611	184	18
		133	56.5/5.2	3/635	100	5
		136	56.5/5.2	15/642	372	24

ULA1_HUMAN	NEDD8-activating enzyme E1 regulatory subunit	133	60.7/5.2	6/635	127	8

VATB2_HUMAN	V-type proton ATPase subunit B, brain isoform	153	56.8/5.6	4/615	191	12
		154	56.8/5.6	9/576	252	14
		155	56.8/5.6	7/637	300	16

VDAC1_HUMAN	Voltage-dependent anion-selective channel protein 1	300	30.9/8.6	7/601	128	18
		304	30.9/8.6	34/536	902	59
		305	30.9/8.6	19/524	319	38

VIME_HUMAN	Vimentin	130	53.7/5.1	2/614	68	6
		161	53.7/5.1	28/626	710	46
		175	53.7/5.1	44/592	1026	63
		178	53.7/5.1	46/621	1306	58
		222	53.7/5.1	3/630	87	6
		228	53.7/5.1	5/664	139	9
		239	53.7/5.1	4/638	139	9
		240	53.7/5.1	5/656	169	12

VINC_HUMAN	Vinculin	25	124.3/5.5	14/578	226	16
		27	124.3/5.5	30/583	643	25

**Table 3:** Flow cytometric analysis of cell surface expression of CD73, CD90, and Annexin A2. Data represent median% (range); *n* = 3 replicate experiments.

Antigen	PDLSC	GF	Keratinocytes
CD73	**99.90**	**99.97**	**0.82**
	(99.4–99.9)	(99.9–100)	(0.78–0.84)

CD90	**99.98**	**99.98**	**1.58**
	(99.8–100)	(99.9–100)	(1.55–1.62)

Annexin A2	**4.48**	**2.92**	**0.86**
	(1.92–7.83)	(2.41–4.66)	(0.86–1.64)
